# The Economic Use of the Medical Profession

**Published:** 1917-12-08

**Authors:** 


### HAY CAMPS.

### The Economic Use of the Medical Profession.

A COUNTRY PRACTITIONER writes:—

There has been in this village for some months past a hay camp consisting of men in khaki, who, whilst not medically fit for active service, have been allotted the task of ricking hay purchased by the Government. The men are in a low category and need a considerable amount of medical attention. For this service, and I presume the method applies throughout the country generally, a civilian doctor has been appointed by Government. In this particular instance, a man who lives between five and six miles away has been given the appointment, although the camp is within a mile of my house. The explanation, I am told by him, lies in the fact that he is paid a fixed sum for every twenty-five men or fraction of twenty-five, and as a camp was started in his neighbourhood before the inauguration of the one near me, he was given the medical charge of the latter in order to save expense. To illustrate, let us assume his camp contains eighty men, and the one near me seventy. Adding these figures together we get 150. On the basis of payment he would be remunerated for six batches of twenty-five. On the other hand, had I been appointed to the camp here my colleague would have to be paid for four batches of twenty-five, and I for three batches. Thus the Government would be the loser to the extent of payment for one batch. Against this, however, is to be placed the fact that a man is taken ill on one of the days on which my colleague does not come over (he comes, ordinarily, three days out of the seven) I can be called in, and may charge the Army for each separate attendance at the usual rates obtaining in the case of men taken ill whilst home on leave.

Whether, and, if so, to what extent the Government save by this arrangement it is difficult to say, but it cannot be very much. And whilst the above imaginary figures have been deliberately chosen in order to show to what extent the Government may lose by giving the appointments to two doctors, the numbers of men to be treated might so happen to be such that there would be no loss. In such a case the Government would lose by appointing only one man, since the unappointed man would still have to be paid the extra fees for separate attendances. Further, the men in the camp near to me have now been billeted in this village for the winter months, which means that a doctor who lives miles away, and who in ordinary circumstances would never, practically speaking, be in the village, is going in and out of the houses of my patients. I am casting not the slightest reflection upon my colleague; but, on general principles, is it fair to bring a man regularly three days a week into another one's preserves, thus familiarising him with another man's patients, and the patients with another doctor? We all know how "a new doctor" appeals to a certain class of patient. The question of waste of petrol might perhaps be raised as being another consequence of a one-sided economy.

